# A Tool for Balance Control Training Using Muscle Synergies and Multimodal Interfaces

**DOI:** 10.1155/2014/565370

**Published:** 2014-05-29

**Authors:** D. Galeano, F. Brunetti, D. Torricelli, S. Piazza, J. L. Pons

**Affiliations:** ^1^Catholic University of Asunciόn, 1394 Asunciόn, Paraguay; ^2^Bioenginering Group, Spanish Research Council (CSIC), 28500 Madrid, Spain

## Abstract

Balance control plays a key role in neuromotor rehabilitation after stroke or spinal cord injuries. Computerized dynamic posturography (CDP) is a classic technological tool to assess the status of balance control and to identify potential disorders. Despite the more accurate diagnosis generated by these tools, the current strategies to promote rehabilitation are still limited and do not take full advantage of the technologies available. This paper presents a novel balance training platform which combines a CDP device made from low-cost interfaces, such as the Nintendo Wii Balance Board and the Microsoft Kinect. In addition, it integrates a custom electrical stimulator that uses the concept of muscle synergies to promote natural interaction. The aim of the platform is to support the exploration of innovative multimodal therapies. Results include the technical validation of the platform using mediolateral and anteroposterior sways as basic balance training therapies.

## 1. Introduction

Balance control is a critcal aspect for the growing elderly population and one of the first rehabilitation goals after spinal cord injury (SCI) and stroke [1].

Generally, balance control rehabilitation consists of the execution of specific movements (sway, inclination) or the adoption of static postures. In some therapeutic scenarios, electrical stimulation is also used to promote the recruitment of the muscles that take part in balance control. Recently the use of robotics devices has been also proposed, without yet generating a significant impact on the clinical practice. In this scenario, computerized dynamic posturography (CDP) is a valuable tool to measure balance control and to assess neuromotor recovery through a rehabilitation process.

In this paper, we present a novel multimodal tool for balance control training of elderly and neurologically injured people. The system makes use of the concept of muscle synergies, in the attempt to achieve a closer and more natural interaction with the central nervous system (CNS) of the patient, possibly resulting in better rehabilitation outcomes.

The document is structured as follows. A detailed introduction on the motivations behind the development of the platform is given in Sections 1.1, 1.2, and 1.3.  Section 2 describes how the paradigm of muscle synergies is applied to postural control rehabilitation. In Sections 3 and 4, the technical details of the system are presented, with special attention to the importance of timing and synchronization between the multiple interfaces and devices used. In Section 5, the results of the technical validation are presented. In Section 6, we discuss the concepts and features of our platform and present our conclusions.

### 1.1. Evaluation of Postural and Balance Control

CDP can be considered as an objective assessment tool of postural control. It allows us to know a subject's ability to integrate information from the visual, vestibular, and somatosensory systems or possible alterations of these systems. It enables the assessment of multiple pathologies that can manifest as loss of balance control, vestibular diseases (Meniere's disease, positional vertigo, and vestibular neuritis among others), or neurological diseases (multiple sclerosis, brain trauma, etc.) [2]. Posturography is usually used to design and implement tailored retraining program balance using visual feedback techniques based on diagnosed sensory deficits and functional capacity of the patient. Besides all these advantages and applications, posturographic systems enable the physician to monitor the evolution of subject treatment and help to evaluate the effectiveness of the prescribed therapy [1, 3].

CDP is based on the idea that the oscillations of the center of pressure (CoP) reflect the postural instability [4]. In line with this hypothesis, the American Medical Association (AMA) has included the monitoring and assessment of CoP trajectories as one of the methods that allows us to objectify deficits or disabilities of postural control. The American Academy of Neurology affirms that CoP monitoring is a useful clinical tool for the analysis of human balance [5]. The CoP is measured by means of force platforms [6], which record forces and moments in the three orthogonal axes. The information is collected by a computer application and used to calculate parameters and indexes from the displacement of the CoP trajectory.

The main drawback of CDP systems is the cost of the equipment. The second major drawback is that there is no agreement in the literature regarding the validity, importance, and relevance of certain postural parameters and indexes obtained by these posturography tools and their relationship with neuromotor disorders.

**Figure 1:**
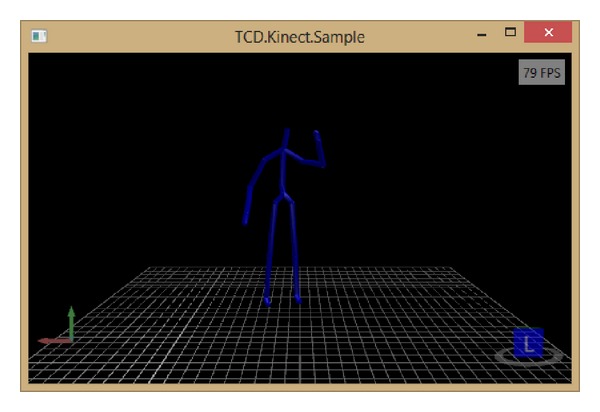
3D skeleton tracking with Kinect.

**Figure 2:**
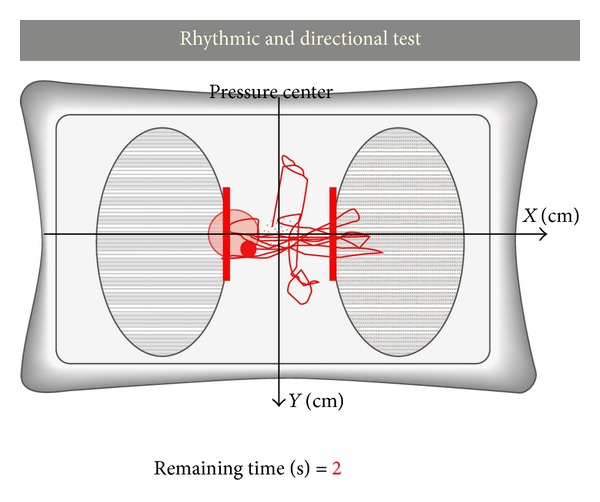
Patient's reference during the rhythmic and directional test.

In the framework of the project HYPER, which is aimed at developing new therapies based on novel neuroprosthetic and neurorobotic solutions, we developed a low-cost posturography and balance training system, composed of inexpensive components like the Wii Balance Board, the Microsoft Kinect, and an electrostimulator. The Wii Balance Board is responsible for obtaining registration of the CoP during testing, whereas the Microsoft Kinect provides online and offline posturography measurements (skeletal tracking). The electrostimulator, called TEREFES [7], provides appropriate muscle stimulation by means of electrical current. All details of the designed posturography system can be found in [8]. Latest changes to this posturography system that are not included in [8] are simplified architecture of the skeleton tracking in 3D with Kinect using  SkeletonPainter3D [9] (see Figures 1 and 2) and user interface changes in posturography software.

### 1.2. Rehabilitation of Postural Control

Current efforts in rehabilitation research are increasingly focused on the integration of neuroscientific knowledge in order to develop new effective means of neurorehabilitation based on a deeper understanding of the human control system. At the same time, the recent advances in low-cost technology permit us to complement the basic rehabilitation protocols with new techniques, such as the Rhythmic Weight Shift (RWS) Test [10], the use of functional electrostimulation (FES) [11], robotic devices according to the assisted as needed (AAN) paradigm [12], and virtual reality systems [13].

The RWS Test consists to follow a target reference that moves sinusoidally in the medial-lateral or anterior-posterior axis. This test is included in most commercial posturography platforms [14–16]. Used as a training, RWS exercises can generate several benefits as improved balance and reduced risk of falls. Positive effects can extend to other tasks as improved gait kinematics and walking and appears to indirectly contribute to the social insertion of the patient in the community [17].

*Functional electrical stimulation* (FES) therapy consists in the application of electrical pulses of current used transcutaneous or surface electrodes, in order to promote the execution of functional movements. In recent years, advances in microelectronics and electrode manufacturing led to the development of more powerful, flexible, and smart electrostimulators [7, 18–20]. New FES systems are capable of handling electrode arrays and enable the implementation of more complex control algorithms to generate dynamic stimulation patterns [21]. Despite these advances, very few solutions reached the clinical practice. The main unresolved problems of FES-based interventions are related to muscle fatigue, coordination of multiple muscular patterns, selectivity of muscles, and functional stability of human-machine interfaces. The controlled application of electrical currents into the body provides both therapeutic and functional benefits. FES can help to avoid atrophy of affected muscles and upper motor neurons and in conjunction with a dynamic activity can improve cardiopulmonary health. There is also growing evidence that FES can improve functional movements, such as running or jumping. Electrical stimulation also has influence on the central nervous system (CNS), probably due to multimodal afferent signals occurring during stimulation. This fact can promote the reorganization of the primary motor cortex [22–24] and change the recruitment techniques of spinal motor neuron pools, [25, 26]. These evidences encouraged the scientific community to use FES as a tool to guide and promote plasticity and adaptation of motor skills to new conditions after stroke or SCI [7].

*Assisted as needed* (AAN) is one of the emerging rehabilitation paradigms currently proposed in rehabilitation robotics [27]. According to this paradigm, the machine is intended to simulate the operation of a therapist during the execution of a motor task, providing assistance only if the patient is not able to execute the movement correctly by him or herself. Most implementations follow fixed profiles in terms of kinematic or dynamic patterns; that is, the patient is asked to follow a fixed path which is assumed to be the correct one. According to [21], this fixed-trajectory approach has two main drawbacks: (i) it does not take into account the specificity of the patient, since the reference trajectory is fixed for all users, and (ii) it ignores the implications of muscle activity within the therapy, by acting only at a kinematic level.

### 1.3. Muscle Synergies and Rehabilitation

The rehabilitation paradigm of the presented platform is based on the recent theory of muscle synergies [28]. From a biomechanical point of view, the human body is a redundant system of many degrees of freedom. The efficient and robust control generated by the CNS is still not sufficiently understood [29]. Recent neurological research hypothesizes that the CNS incorporates a library of motor activation modules in order to exert specific and common motor tasks by contributing to the synchronized activation of different muscles. These modules are called muscle synergies, and their combination can lead to more complex and functional muscle activation patterns at the spinal level, while maintaining a relatively simple control at higher centers.

**Figure 3:**
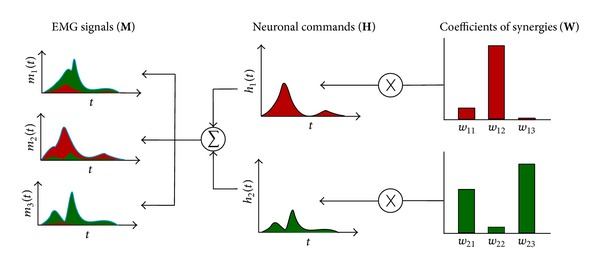
Muscle synergy concept. Each neural command activates a specific synergy with a factor *h*_*j*_(*t*), which may be a function of time or the type of movement. Therefore, muscle activations are a weighted average of the activations of each synergy [27].

Mathematically, muscle synergies can be expressed by the following equation, which describes the activation of a muscle *m*_*i*_(*t*):

(1) $$\begin{array}{c} m_{i}\left(t\right)=\overset{K}{\underset{j}{\sum }}h_{j}\left(t\right)w_{ji}, \end{array}$$

where *m*_*i*_(*t*) is the time function of muscle activation (EMG signal) for muscle *i*, *w*_*ji*_ is the coefficient of *j*th synergy to the *i*th muscle, *h*_*j*_(*t*) is the temporal function of the neuronal command *j*, and *K* is the number of synergies. This concept is shown diagrammatically in Figure 3.

In its complete form, **M** = **H** × **W**, where **M** is a matrix of 1 × *N* (*N* muscles), **H** is a matrix of 1 × *K* (*K* neural modules), and **W** is an array of *K* × *N*.

This process of decomposition and distribution of muscle activation reduces the computational requirements of motor control and possibly influences the learning of new motor tasks [30]. According to this hypothesis, the brain recruits muscle groups (synergies) in spite of controlling individual muscles independently. It has been demonstrated that the composition of muscle synergies depends on the motor task and can be affected by neuromotor pathologies [5]. According to [27], taking a set of synergies as a reference for a rehabilitation task may have two innovative potential effects:

- improving neural plasticity, since the therapeutic action is located at the level of muscle activation, which is closer to the CNS with respect to kinematics;
- adapting to subject-specific kinematics constraints, since synergies mainly depend on the functional goal and not on biomechanical constraints.

These potential effects are motivated by three key properties of muscle synergies. First, muscle synergies have been found to be consistent among subjects despite the precise kinematic trajectory [31, 32]. Second, they can be trained and are prone to change if task conditions change [33–35]. Finally, they somehow codify functional movements, so they can be used to train specific rehabilitation targets [36].

## 2. Synergies and Balance Control Training Based on Sway Movements

According to [5], the application of the concept of muscle synergies in clinical settings is twofold. In the* diagnosis* of neuromotor disorders, it may provide access and an overall view of motor control information at a higher level than muscular activity. In the* rehabilitation* field, it can help in the effective design of better training and rehabilitation therapies, for instance, in combination with an FES system. In order to define a reference set of synergies, a series of experiments were conducted to study healthy subjects during RWS in the mediolateral (ML) and anteroposterior (AT) directions [5].

**Figure 4:**
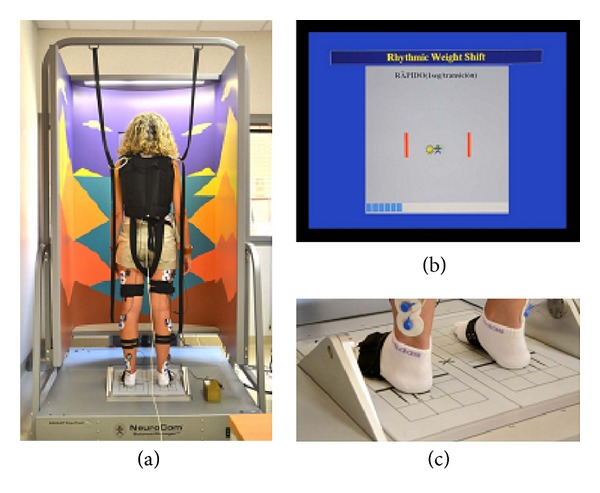
On the left, there is an instrumented subject on the NeuroCom Smart Equitest (Oregon, USA). On the top-right, there is the visual interface of the patient. It represents the current exercise, indicating with a stylized dark man the current position of the CoP of the patient and with a yellow sun the target position of the exercise. On the bottom-right, there is a detail of the dynamometric platform the forces applied by the user to determinate CoP position [5].

This experiment was designed to measure the trajectory of the CoP of 6 healthy subjects (3 men and 3 women), during the rhythmic postural sway in the ML and AT directions. The CoP was measured using a CDP platform, the Neurocom   Smart Equitest, which is depicted in Figure 4. The patient's visual reference was replaced by an auditory reference, a digital metronome, indicating the expected frequency of the movements. This change was motivated by the intention of increasing the VAF of the synergies [5]. During the execution of this test, electromyographic signals (EMG) were acquired using a 16-wireless channel electromyograph (ZeroWire). Three sway frequencies were defined (low = 0.167, medium = 0.25, and high = 0.5 Hz). The sway frequencies obtained by the patients are shown in Table 1. The algorithm NNMF (nonnegative matrix factorization) was used to extract the values of **H** and **W**. The EMG signal reconstructed using **H** and **W** (1) has then been compared with the original one. The quality of the reconstruction was expressed with the variance accounted for (VAF) [5].

**Figure 5:**
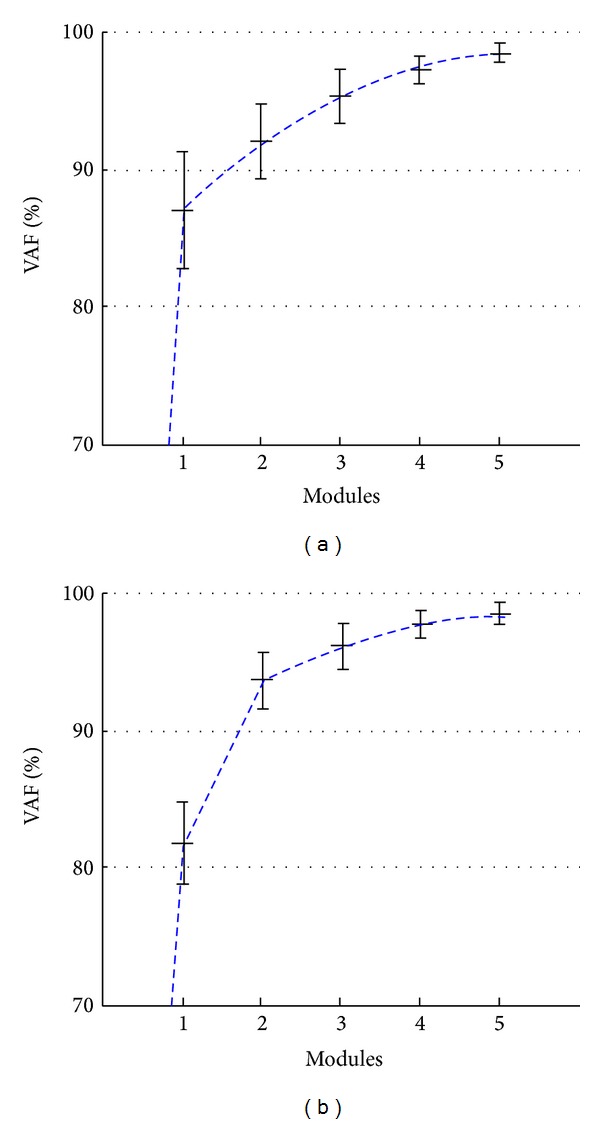
Variance accounted for (VAF) as a function of the number of modules during the execution of mediolateral (a) and anteroposterior (b) sway movements [5].

Results revealed that two synergy modules are responsible for more than 90% of VAF (Figure 5) for all subjects in all conditions. This study showed evidences that support the existence of consistent modular control in healthy volunteers while doing lateral sway movements. Similarities can be seen in terms of number of modules, composition of synergies, and time-varying activations. Two modules represent most of the variability of the EMG for all subjects. Coefficient values are similar for different frequencies. This fact suggests that the muscle coordination and muscle synergies are not much influenced by the movement speed.

The comparison between subjects suggests high similarity in muscle synergies modules. However, a dedicated analysis should be performed to confirm this hypothesis. With the synergies modules found for these tasks, we proceeded with the development of a multimodal tool for balance control training.

## 3. Training Platform

**Figure 6:**
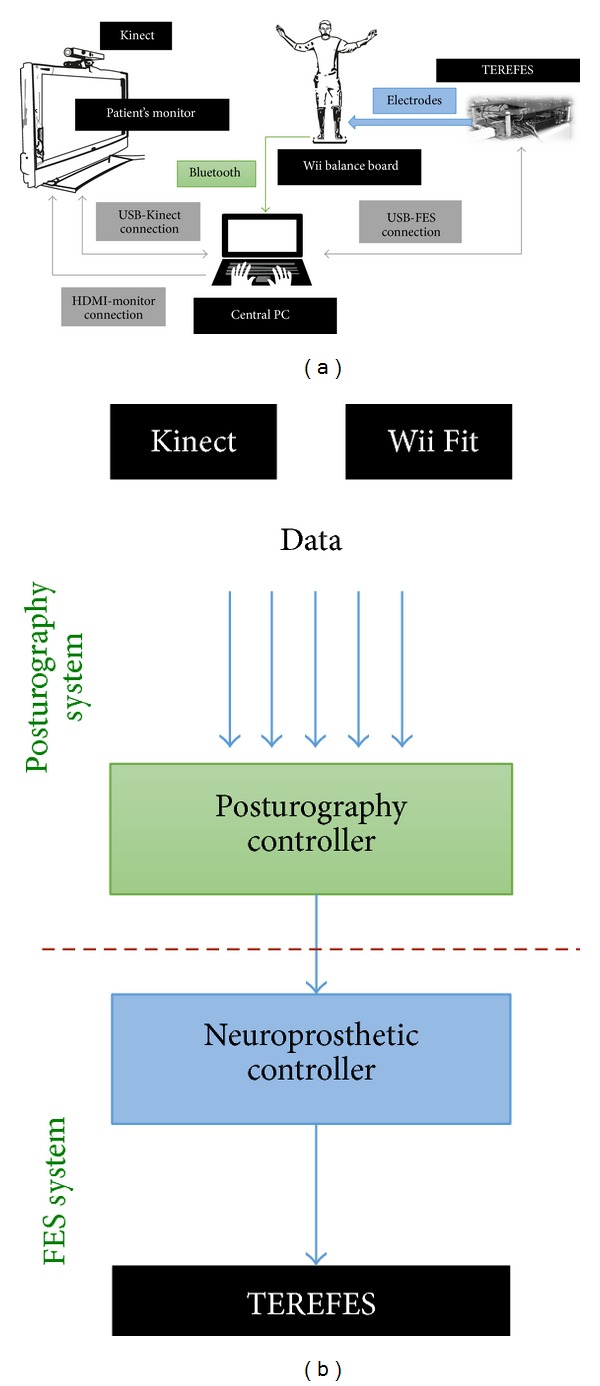
(a) Proposed platform architecture and (b) functional description diagram including the different components: the posturography system (balance control assessment) and the FES system (balance control training and rehabilitation).

In this section, we present a low-cost training platform for balance control. The contribution of this tool relies on the use of muscle synergies and multimodal interfaces. The system is based on a previously developed low-cost posturography platform [8]. It includes inexpensive components, such as the Wii Balance Board (WBB), the Microsoft Kinect, the TEREFES electrostimulator [7], and a central PC running a synergistic control algorithm driven by the position of the CoP of the subject. The kinematic information of the CoP is retrieved by means of the Microsoft Kinect and the WBB. This set of interfaces enables the development of close and open loop training tasks, using different type of interaction and information. The system includes a visual feedback (monitor) and an auditory reference (digital metronome). The proposed architecture is shown in Figure 6.

### 3.1. Wii Balance Board

The WBB is an input device manufactured by Nintendo. It is a wireless device that communicates with the Wii console using the Bluetooth standard. It has a dimension of 45 × 26.5 cm and contains four pressure sensors located in each corner to measure the force in the vertical direction. The performance of WBB has been already studied in the literature [37–40], and the general conclusions are that the WBB can replace conventional force platform in slow range movements (0.01 Hz–10 Hz). These movements do not require a higher resolution. The platform specifications fulfill all requirements for our application and studies.

#### 3.1.1. Connectivity

A specific study to analyze the connection jitter of the received data by the WBB was carried out in the literature [8]. The result shows that the sample frequency responds to a nonparametric probability distribution with a mean value given at 100 Hz. This result matches the sampling period specified by the manufacturer. In the mentioned study [8], it is also shown that the probability that the sample frequency being greater or equal than 50 Hz. is 94.02%. Test condition has been fully described in the paper.

#### 3.1.2. Accuracy and Reliability

Consistent documentation about the force and center of pressure (CoP) accuracy and reliability of the WBB is not available. However, [41] reported that the force measurements total uncertainly was within ±9.1 N and that the CoP location was within ±4.1 mm between different WBBs [41]. They also found that the measurement repeatability of both parameters are ±4.5 N and ±1.5 mm with each WBB. They suggest that the WBB may be useful for low-resolution measurements but should not be considered as a replacement for laboratory-grade force plates.

In golden standard commercials force plates, sensors register forces and moments in 3D. These force plates are accurate and durable and recordings are independent of temperature and stable along time. The disadvantages are low portability due to mass (10–45 kg) and mounting requirements and their cost (US$ 15000–20000). Commercial low-cost platforms usually have the same quantity of pressure sensor, as WBB does. The difference between these systems is the resolution accuracy and possibility of 3D force measurements. However, for CoP assessment, a pressure platform is enough and there is no need for a 3D dynamometric platform. Typically, commercial platforms have a resolution less than 0.2 mm. According to [42], the CoP resolution of the WBB is approximately 0.5 mm.

The estimation of CoP using WBB has a drawback. The shear forces and moments cannot be taken into account. As a consequence, these additional forces are neglected and problems may arise when trying to assess dynamic movements or static forces applied in the horizontal plane.

#### 3.1.3. Library

A library called Wiimotelib permits us to link the WBB system with other Wii accessories and is programmed in C# language. Using this library we can connect with the device and get all WBB data (CoP displacement, battery status, etc.) as a burst of events that the WBB sends continuously to the PC. There is also a community called Wiibrew [43] that provides support for developers regarding these libraries and the devices.

### 3.2. FES System

The TEREFES was proposed within the framework of the TERERE and HYPER projects [7]. The TEREFES electrostimulator provides up to 32 stimulation channels driven by controllable, stable, and close loop current sources. In addition, the system is portable and flexible. It is powered by 4 AA batteries and includes a USB communication interface (1 Mbps) that allows its configuration via external software. Monophasic and biphasic stimulation signal can be obtained across the 32 available channels. These channels are divided into two independent groups of 16 channels each that can be stimulated simultaneously. The TEREFES has a current range of 0–150 mA, pulse width of 0–5 ms, high frequency stimulation of 100 Hz, and maximum stimulation voltage of 250 V.

A synergistic FES controller was already proposed in [44]. Following these concepts, an upgrade of firmware of TEREFES has been done in order to meet technical specifications required by the experimental validation. Two parameters could be changed while stimulating muscle synergies: amplitude and pulse-width. All the changes in the parameters can be performed while the stimulation is running. This feature allows us to develop dynamic stimulation algorithms.

## 4. Timing and Synchronization of the Multimodal Interface

The proposed platform can be a seen as a control system, characterized by a strong human-machine interaction. This interaction, specifically the one related to FES, is control-oriented, meaning that FES is prone to generate motor effects in users. In many cases, a hard real-time system would be the best solution for this type of application. However, in this scenario, where many different and distributed technologies are combined together, the development or use of a real-time architecture may compromise the use of these low-cost components.

**Figure 7:**
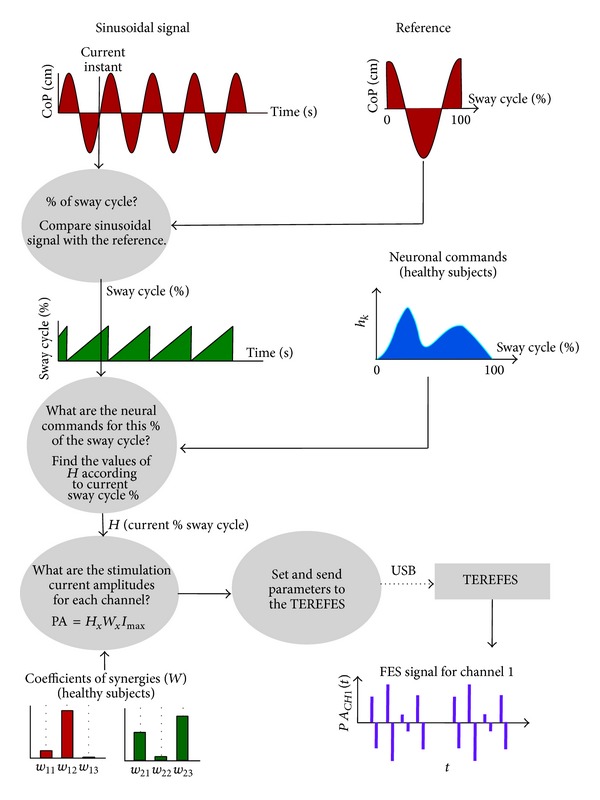
Functional diagram of the synergistic controller implemented in the PC.

The system was developed for training balance control during ML and AP sway movements and works as follows. Prior to the training session, four variables should be defined (i) **H** and **W** (activation and synergy) matrices, as obtained from human experiments; (ii) the mapping between the columns of **W** (muscles) and the TEREFES' channels (0–31); (iii) the maximum current for each channel, **I**_max⁡_; and (iv) the frequency of the sway movement. According to the selected frequency, the system computes a reference signal, namely, the CoP reference trajectory, which the subject should follow during the training session. The system uses a digital metronome to help subjects to synchronize with this reference signal during the session. The system continuously calculates the neural command for every point of the CoP reference trajectory and, according to this value, it computes corresponding current pulses to be applied to each muscle at a given time. All this process is illustrated in Figure 7.

The synergistic controller is responsible for calculating the muscle activations as a function of CoP coordinates and for controlling the currents to be applied to muscles through the TEREFES. So far, it works in an open loop manner. This means that the synergistic controller only considers the CoP reference to calculate muscles activations. The measured CoP provided by the WBB is just used to provide visual feedback to the user. The synergistic controller runs in the PC. It is an application developed in C# Visual Studio 2012. In order to calculate the desired muscle activations, the controller uses synergies (**W**) and activation coefficients (**H**) obtained during ML and AT experiments previously described. Because of the cyclical nature of the sinusoidal reference, the neural commands were calculated as a function of the percentage of the sway cycle. Each point of the sine wave corresponds to a certain percentage of the sway period and also to a specific neural command value. Thus, there is a direct mapping between current position value of CoP and neural commands. Specifically, the neural commands were calculated with a variation of 1% of the sway period, getting a total of 100 samples per cycle.

The system assumes that the patient starts at a certain point of the sway cycle, usually where the CoP trajectory is zero, meaning approximately 75% of the sway cycle, as shown in Figure 7. It is further assumed that a therapist or a robotic system ensures that the patient follows the simulated reference. The application allows us to compare online the measured CoP trajectory by the WBB with the one generated by the system. Thus, the therapist and the user can know how synchronized the current movement and the reference signal are.

The synergistic controller has to keep a precise timing in order to control effectively and periodically the TEREFES stimulation parameters. This is achieved by using the Stopwatch class of C#. This class allows us to measure execution times with high precision. Each time this timer expires, the synergisitc controller calculates the percentage of the sway cycle based on the current amplitude of the CoP trajectory. The result is transformed again into the correspodding stimulation parameters that are afterwards transmitted to the TEREFES. Once the TEREFES receives new modulation parameters, it immediately updates its parameters.

Since the controller is running over non-real-time operating systems and communicates with the TEREFES using also a non-real-time communication technology (USB), the systems take advantage of real-time TEREFES hardware to implement a timing monitor to verify that the stimulation parameters are updated periodically in a precise manner. Thus, the systems know when the timing requirements are and can stop the control/stimulation signals.

The system also includes a second synchronization tool that monitors the differences between the reference local CoP and the real user CoP, so that user and therapist can train prior to the synergistic stimulation.

The synergistic controller has several other functions, as summarized in the following part.

1. *Capture*. It continuously receives WBB samples at an average frequency of 100 Hz.
2. *Show*. The program displays the trajectory of CoP, the sampling period of the WBB, the current percentage of sway cycle, and the precise timing of TEREFES configuration.
3. *Save*. The application saves all the data that has been acquired, calculated, or received, in MAT files that can be later exported to MATLAB for further analysis.
4. *Debugging*. The application has implemented a serial terminal, allowing the user to send and receive TEREFES commands.
5. *Configuration*. The application allows the configuration of several parameters, such as the frequency of the stimulation of the TEREFES, the refresh rate of synergies, test duration, the mode of operation of the TEREFES (single burst or continuous mode), and the amplitude and frequency of the sine wave reference.

## 5. Results

In this section, we present the preliminary results of our platform. We carried out two experiments. The first one was aimed at verifying the performance of the system in terms of timing, focusing in particular on the limitations of the non-real-time architecture running on Windows. The second experiment was designed to validate the proposed system with the ultimate goal of generating correct FES timulation patterns according to the concept of muscle synergies. The following sections detail these two experiments.

### 5.1. System Performance

There are two fundamental parameters of the system. The first one is the frequency stimulation of the TEREFES (*F*_*t*_). The device must continuously stimulate at a certain frequency (usually in the range of 40–50 Hz). This frequency is fixed and controlled autonomously within the TEREFES. The second important parameter is the refresh rate of muscle synergies (*F*_*s*_), which should be lower than *F*_*t*_ but large enough to sample every variation of neural commands during the sway cycle. From previous experiment, we observed that there is high correlation between the frequency of the reference sine wave and the frequency of neural commands [5]. Considering that voluntary postural sway frequency usually lies below 0.5 Hz, a synergy frequency of 5–10 Hz may be enough for these movements.

Different tests of 50 seconds duration were performed. In each test, the TEREFES frequency was set to *F*_*t*_ = 40 Hz (implies a period *T*_*t*_ = 1/*F*_*t*_ = 25 ms). Four frequencies for synergy modulation have been chosen: *F*_*s*_ = {1,5, 10,20} Hz meaning that synergy update period was *T*_*sE*_ = {1000,200,100,50} ms, respectively. The reference sine wave was set at a frequency of 0.1 Hz for all tests.

We defined the period of synergies (*T*_*s*_) as the period which take to the operating system to refresh the new value to the TEREFES. In fact, *T*_*s*_ = 1/*F*_*s*_, where *F*_*s*_ is the frequency of synergies. Given the random nature of the period of synergies (the controller is running on a nonreal operating system), we analyze the performance of the system in all listed conditions. The values of the random variable *T*_*s*_ were measured in the C# application, using the methods of the class Stopwatch and the TEREFES with a 500 *μ* timer accuracy. The results were analyzed with the Distribution Fitting Tool in MATLAB R2011a.

Since *T*_*sE*_ = {1000,200,100,50} ms are the expected values (configured) of the synergies period, we analyze the probability that the random variable *T*_*s*_ is less than or equal to *T*_*sE*_ ± *T*_*t*_, where *T*_*t*_ = 25 ms for all conditions. That is, how likely *T*_*s*_ is below (or postponed to) its expected value during stimulation.

This tolerance window for *T*_*sE*_ is acceptable if we analyze the behavior of the FES system. The FES system generates a pulse train for *N* stimulation channels (1). Stimulation of each channel (current pulse) occurs every *T*_*t*_. If a new synergy value is obtained, the stimulation parameters of every channel are updated. However, if this update takes place during an on-going stimulation, only those channels, which were not stimulated yet, are affected by these new values. This behavior allows us to establish a tolerance of stimulation period, because it is not possible to establish perfect synchronization between non-real-time systems. Also because the frequency of the TEREFES is much higher than synergies frequency, changes during a period of TEREFES are not relevant in obtaining the envelope of the stimulation signal.

**Figure 8:**
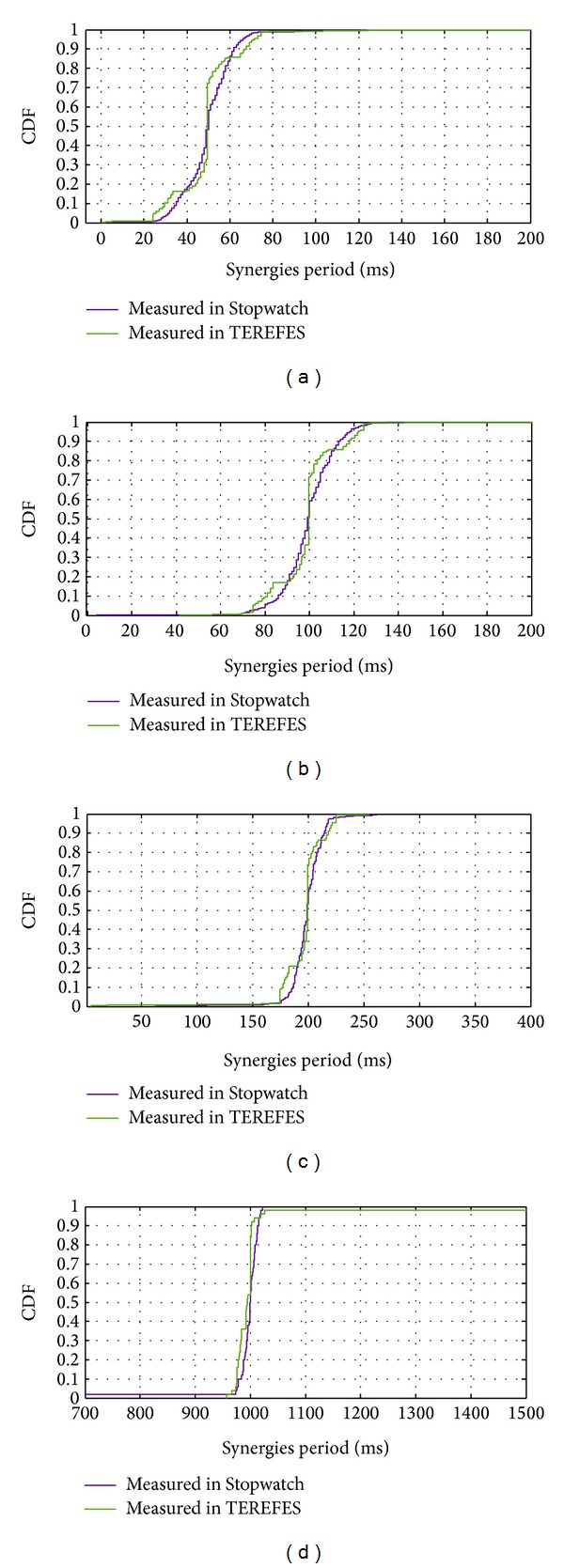
Cumulative distribution function of synergies period for (a) *T*_*sE*_ = 50 ms (*F*_*sE*_ = 20 Hz), (b) *T*_*sE*_ = 100 ms (*F*_*sE*_ = 10 Hz), (c) *T*_*sE*_ = 200 ms (*F*_*sE*_ = 5 Hz), and (d) *T*_*sE*_ = 1000 ms (*F*_*sE*_ = 1 Hz).

The results are shown in Figure 8. The high probability (>95%) that the update of the synergies occurs before the expiry of a further period of TEREFES and the low probability (<3%) that the update occurs before the stimulation period represent a metric for the good performance of the platform. High similarity between the results obtained with TEREFES and Stopwatch is also observed.

Two random variables affect the accuracy of the measures from TEREFES and Stopwatch: the shipping time of the serial data and the time that elapses until the TEREFES updates its parameters with the new values received.

**Figure 9:**
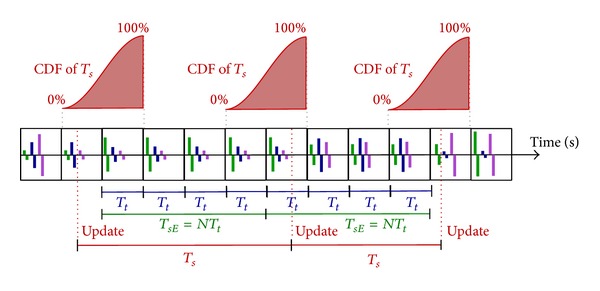
Stimulation patterns for three channels. CDFs are displayed around the expected synergies period (*T*_*sE*_).

The results of the experiments are summarized in Tables 2 and 3. For all frequencies, a concentration greater than 95% of the density of the samples around *T*_*sE*_ ± *T*_*t*_ was observed. This implies an uncertainty of a window of stimulation (*T*_*t*_) around the expected value (right and left). This result is explained graphically in Figure 9.

### 5.2. Reconstruction of EMG Envelopes

This section shows the next validation step aimed to demonstrate that the stimulation signal provided by the TEREFES produces similar muscle contractions as those obtained by the synergistic neural commands, as observed in human experiments.

The validation experiment can be divided into the following steps.

**Figure 10:**
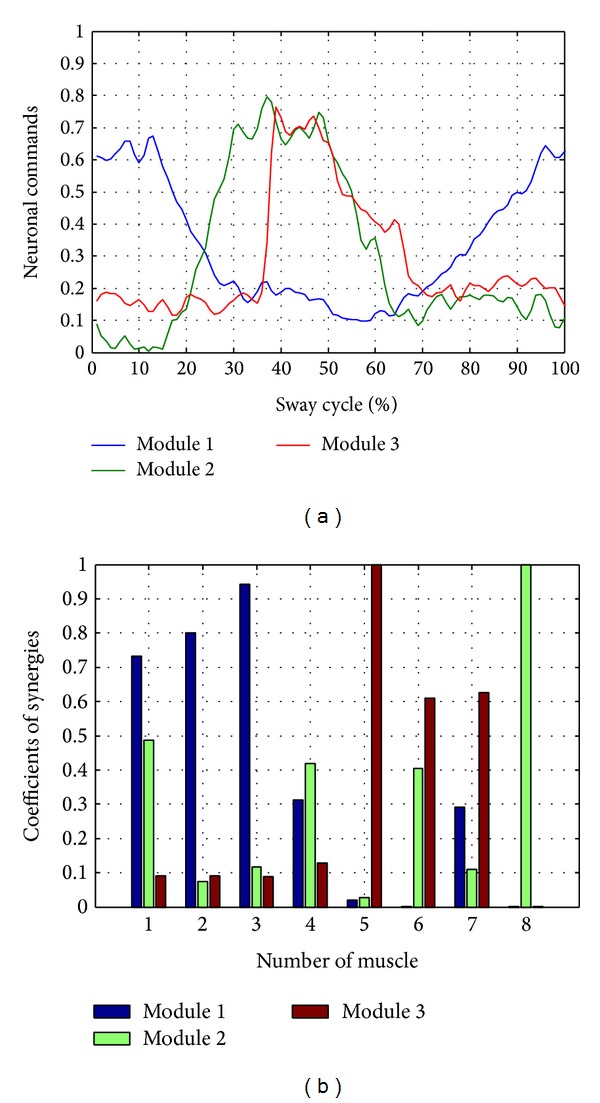
(a) Neural Commands. (b) Coefficients synergies simulated in MATLAB.

1. *Simulation of* **H** *and* **W**. Neuronal commands and synergies have been simulated in MATLAB (Figure 10) and then loaded into the application as MAT files. For this test, three neural modules and 8 stimulation muscles were considered.
2. *Configuration of Parameters*. A sinusoid reference with an amplitude of 10 cm and 0.1 Hz frequency was used. Stimulation was performed at a frequency of 40 Hz, and the synergy frequency was 20 Hz. Test duration was set to 50 seconds.
3. *Data Collection*. During stimulation, the TEREFES monitors the timing of the overall system. To do this, the device measures the elapsed time between consecutive reception of new stimulation parameters sent by the synergistic controller. The measured value is sent back to the controller. These values are stored in the application in dynamic lists.
4. *Data Analysis*. When the test is complete, we can export all the data collected in MAT files. They include synergies period measured by Stopwatch and TEREFES, WBB data arrival periods, values of CoP displacements, and a diferent kind of vector which measures the point in time when events occur in the execution time. For instance, we use Stopwatch class timers to know (i) when the configured execution time is finished, (ii) when a new data from the WBB arrive, (iii) when a new frame is sent to the TEREFES; and (iv) when the new synergy period value measured by the TEREFES arrives. All of these data were previously recorded in dynamic lists when the test was run.

Using temporary values (when we send a new frame to the TEREFES), it is possible to simulate in MATLAB the outputs obtained with the FES system during the test. In this simulation we considered the resolution of the TEREFES, which means that the TEREFES output simulated is the same as that generated.

**Figure 11:**
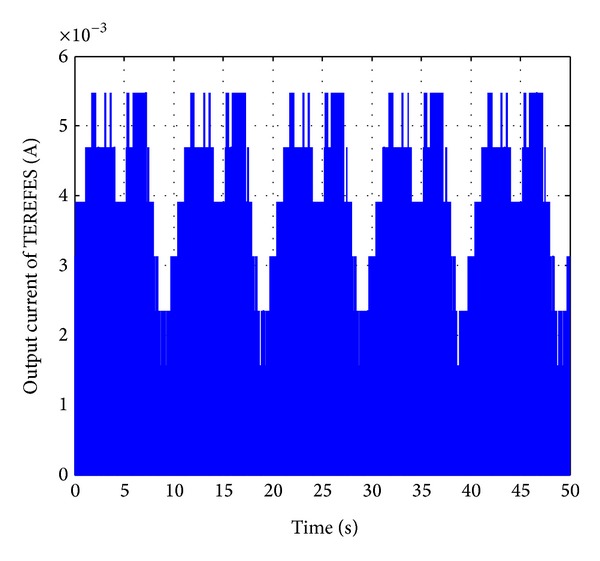
TEREFES output current for muscle 1. The signal is modulated in amplitude according to **M** = **H** × **W** × **I**_max⁡_ during 50 seconds.

In Figure 11, the output generated by the TEREFES for a single channel (corresponding to muscle 1) is shown. The output signals are modulated in amplitude according to the theoretical values of reconstructed EMG envelopes obtained for healthy subjects (according to (1)). To retrieve the EMG envelope signal, a low pass filter is applied to the TEREFES outputs. The butterworth filter was designed in MATLAB, and the filter parameters were set, as shown in Algorithm 1.

**Figure 12:**
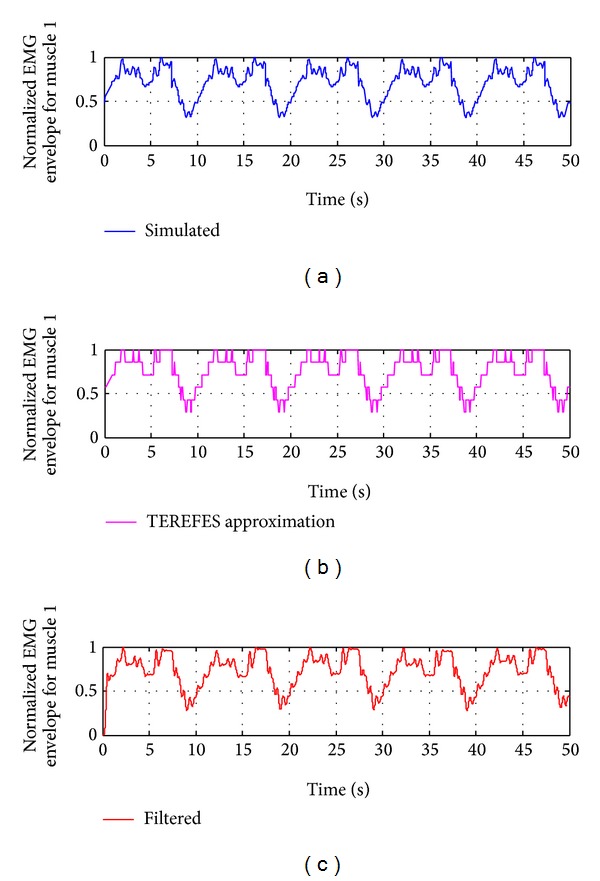
Comparison between normalized EMGs envelopes for muscle 1.

The filter represents roughly the low-pass behavior of the skin-muscle system. The resulting signal of the designed filter is shown in Figure 12. The theoretical EMG envelope for muscle 1 (according to the (1)) is plotted in blue, the envelope signal for the TEREFES output (adjusted to resolution of 782.7*μ*A) is in magenta, and the envelope obtained by filtering the TEREFES signal is in red. The similarity between the first and last signals allows us to demonstrate that the stimulation patterns obtained with the synergistic controller represent in a good manner the muscle activation used previously to obtain these stimulation patterns.

## 6. Discussion

In this work, we presented an innovative balance training system, which combines postural analysis with synergistic electrical muscle stimulation. The experiments presented here were mainly focused on testing the timing and synchronization performance of the system. Timing is a particularly crucial aspect, because the proposed system is made of commercial off-the-shelf technology, which prevents them from having real-time performance. Several efforts were dedicated to get the best timing performance of the system. For instance, two timing and synchronization monitors were implemented in a distributed way, to robustly verify that timing is correct during training sessions. The system succeeded in providing synergistic EMG profiles, but only in an open-loop fashion.

Closed loop control might be needed depending on the rehabilitation task strategy. For example, the system could stimulate according to the current user's CoP and not as a function of a predefined reference. In such closed-loop configuration, a better and deeper knowledge regarding the effective sensing-to-actuation time is needed. According to the literature, this time can be approximately 100 ms, which lies in the same order of magnitude of the system control frequency imposed by the different modules (WBB and TEREFES). This immediately shows that a close loop strategy would be very challenging, at least based on a sample to sample feedback.

Regarding the multimodal interfaces, it is worth to mention that the human-machine system communicates through kinematic, CoP, auditory, visual, and electric signals. The potential use and further impact of this multimodal interfaces have not been explored yet. Despite these aspects, an important outcome of this work is the development of a flexible and powerful tool for the assessment and training of balance control using off-the-shelf technologies.

The potential impact of the introduction of novel concepts and platforms for balance training is huge but still not completely defined. The use of muscle synergies paradigm as a basis for the rehabilitation paradigm represents the most scientific innovative aspect behind this work and still needs to be deeply explored. In order to test this approach, new tools are needed, and the one here proposed may be one of them. The objective of this paper is to provide the technological tool to explore this rehabilitation approach, but further trials are needed to define its effectiveness.

The impact of FES on internal neuromotor mechanisms is still far from being understood, and its use in rehabilitation may vary depending on the therapy. There is evidence of cause-effect relationship, but no extensive description is provided in the literature. The therapy based on electrostimulation can be either applied in an efferent way (mostly functional) or afferent. For both types of electrostimulation, devices are the same, and the difference is mainly in the amplitude of the current signals. In this paper, we always mention the stimulation as a functional one, that is, FES. While the developed system supports both types of stimulations (afferent or efferent), the stimulation profile is another open research variable to be explored.

## 7. Conclusions

In this paper, we have presented a multimodal low-cost tool for training human balance control following a muscle synergy theory scheme. The system consists of a combination of off-the-shelf technologies such as the Wii Balance Board and the Kinect, an electrostimulator and a software application running in a PC.

In this work, we focused on mediolateral (ML) and anteroposterior (AP) voluntary postural sways. For this purpose, the synergy modules described in [5] were used as an input of the system. Based on this information, the system stimulates the muscle synergistically.

The synergy-based controller architecture is based on three non-real-time elements (a) Wii Balance Board; (b) the operating system; and (c) the TEREFES (FES system). The interaction between these off-the-shelf systems was studied and evaluated in this paper. The performance results showed in this paper are expressed in terms of the probability distribution function of timing and synchronization among components. The outcomes of these studies were as follows.

- The system can stimulate with a frequency up to 50 Hz. This frequency range meets the requirement of FES control system available in the scientific literature [7].
- The system can handle and modify while running the stimulation frequency in range of 1–20 Hz. These frequencies are fast enough for balance training since it is a slow movement. The highest frequency of the sinusoidal reference trajectory is 0.5 Hz (sway movement).
- The uncertainty of synergy sampling/update period is approximately ±25 ms (elapsed time between sampled reference trajectory and updated stimulation pulses). It was also demonstrated that the envelope reconstruction is not affected by this uncertainty.

Despite non-real-time performance, the tool showed an acceptable timing and synchronization among modules. These operation ranges are enough for these training scenarios. To get a better online feedback regarding these issues, a monitor of real-time performance was implemented to measure the timing and synchronization between modules.

The system uses multimodal interfaces to get kinematic and CoP information of user and to provide auditory, visual, and electrical signals to the user and the therapist. The interface also includes important features for training purposes, like online graphs of signals, data session storage, and a configuration interface to tailor training for each user.

Future work will be focused on the application of the proposed platform in clinical settings. In particular, preclinical studies in neurologically injured people, for example, spinal cord injured and stroke patients, will be addressed to evaluate the rehabilitation potential of synergistic FES.

## Figures and Tables

**Algorithm 1:**
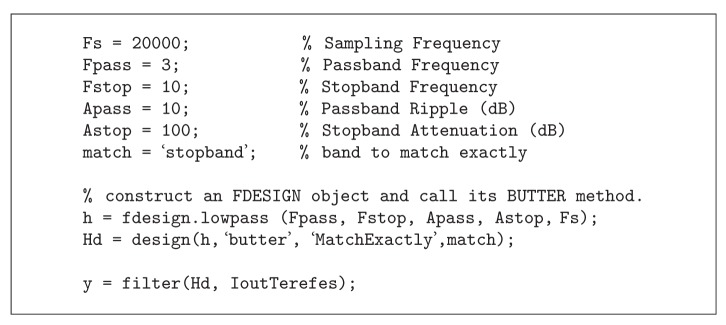


**Table 1:** Target and mean sway frequency of CoP for all subjects. Values are expressed in Hz [5].

Target frequency	0.17 Hz	0.25 Hz	0.5 Hz
Measured frequency (ML)	0.18 ± 0.03	0.26 ± 0.04	0.47 ± 0.11
Measured frequency (AT)	0.18 ± 0.03	0.25 ± 0.04	0.46 ± 0.12

**Table 2:** Probabilistic analysis of synergy update period measured in the TEREFES. *T*_*t*_ = 25 ms and *T*_*sE*_ = 1/*F*_*sE*_.

Parameters	Frequency *F*_*sE*_			
	20 Hz	10 Hz	5 Hz	1 Hz
*P*(*T*_*s*_ ≤ *T*_*sE*_ + *T*_*t*_)	98.87%	99.19%	97.99%	98%
*P*(*T*_*s*_ ≤ *T*_*sE*_ − *T*_*t*_)	0.82%	1.41%	0.8%	6%

**Table 3:** Probabilistic analysis of the synergy period update measured in C# with Stopwatch. *T*_*t*_ = 25 ms and *T*_*sE*_ = 1/*F*_*sE*_.

Parameters	Frequency *F*_*sE*_			
	20 Hz	10 Hz	5 Hz	1 Hz
*P*(*T*_*s*_ ≤ *T*_*sE*_ + *T*_*t*_)	98.88%	98.39%	98%	99.99%
*P*(*T*_*s*_ ≤ *T*_*sE*_ − *T*_*t*_)	0.71%	2.6%	1.6%	2%
